# Radiotherapy for Epstein-Barr Virus-Positive Mucocutaneous Ulcer at the Lower Leg Skin: A Case Report

**DOI:** 10.7759/cureus.30936

**Published:** 2022-10-31

**Authors:** Nobuteru Kubo, Naoko Okano, Hidemasa Kawamura, Akihiko Uchiyama, Tatsuya Ohno

**Affiliations:** 1 Department of Radiation Oncology, Gunma University Graduate School of Medicine, Maebashi, JPN; 2 Department of Dermatology, Gunma University Graduate School of Medicine, Maebashi, JPN

**Keywords:** lymphoproliferation, electron beam, ulcer, ebvmcu, radiotherapy

## Abstract

Epstein-Barr virus-positive mucocutaneous ulcer (EBVMCU) is a rare lymphoproliferation and a relatively benign condition. Although the condition can be cured without treatment, some cases require chemotherapy, resection, or radiotherapy. However, there are no established standards regarding the dose and schedule of radiation therapy. We present the case of a 44-year-old female with a history of living donor kidney transplantation who developed EBVMCU in the right lower leg after 23 years. She did not improve with conservative therapy and was treated with low-dose radiotherapy (4 Gy in two fractions) to the EBVMCU on the lower leg skin. The patient achieved complete control after one year without toxic effects. This case report provides evidence that low-dose radiotherapy is a potentially effective treatment for EBVMCU in patients who do not improve with observation or by decreasing immunosuppressive therapy.

## Introduction

Epstein-Barr virus (EBV), also known as human herpesvirus 4, is widespread worldwide and is carried as a latent asymptomatic infection. EBV is associated with B-cell lymphoproliferative disorders [1-2]. Epstein-Barr virus mucocutaneous ulcer (EBVMCU) is a recently recognized pathological entity that was placed under the category of mature B-cell neoplasms in the 2017 revised 4th edition of the World Health Organization classification of tumours of haematopoietic and lymphoid tissues [3]. EBVMCU is a rare lymphoproliferation with localized, circumscribed, shallow mucosal or cutaneous ulceration in the oropharyngeal mucosa, skin, and gastrointestinal tract. It is characterized by a mixed hematolymphoid infiltrate with Hodgkin-like morphologic and immunohistochemical features. EBVMCU is a relatively benign condition with a self-limited disease course. A conservative approach, such as observation or decreasing immunosuppressive therapy, is the first choice of treatment. The ulcer may regress after the reduction or discontinuation of immunosuppressive therapy without requiring additional treatment. Some cases require more aggressive treatment, such as chemotherapy, resection, and radiotherapy [4-6]. However, there are no established standards of treatment for this disease, and the optimal dose-fractionation regimens for radiation therapy remain to be established [7]. In this case report, we share our experience with the use of low-dose radiation therapy for the treatment of EBVMCU.

## Case presentation

**Figure 1:**
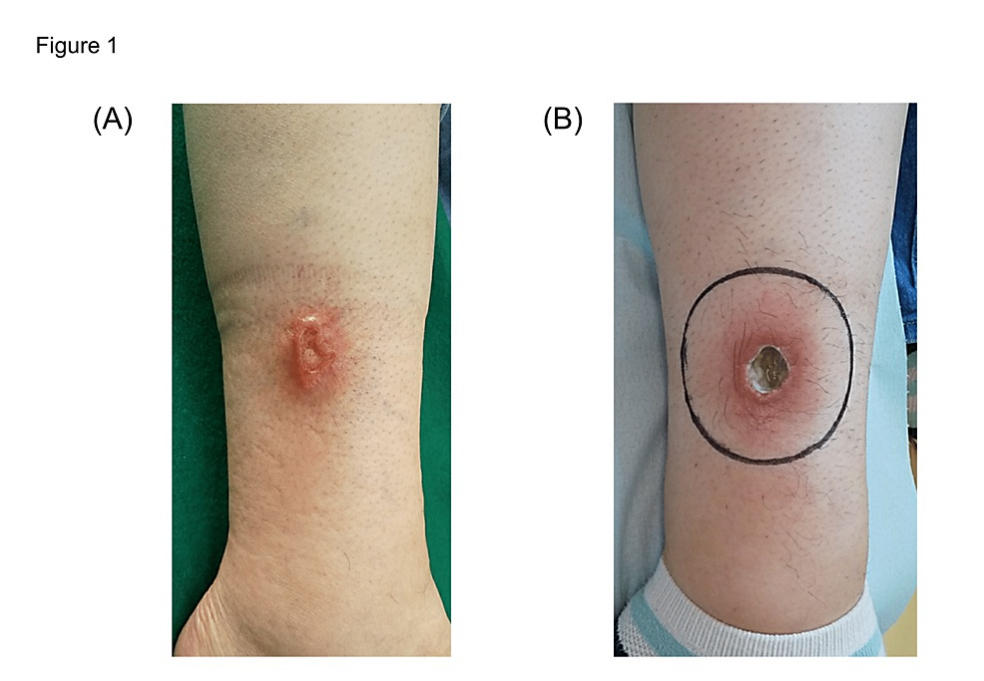
Clinical photograph of the ulcer on the skin of the right lower leg at two months before treatment (A) and electron irradiation field (B). The treatment field is shown as a black circle.

A 44-year-old female presented with a 2 cm ulcer (5 mm in depth) in the right lower leg. The patient had undergone dialysis 34 years before this presentation due to bilateral hypoplastic kidneys and membranous proliferative nephritis; she underwent living donor kidney transplantation 23 years ago. The patient was treated with immunosuppressive drugs (corticosteroid, mycophenolate mofetil, and tacrolimus) as maintenance immunosuppression. At one year and six months prior to radiotherapy, the patient developed erythema on the right lower leg. Topical steroids were prescribed for two months, but the condition worsened (Figure 1A). No other skin ulcerative lesions were observed, and there was no lymphadenopathy, organomegaly, or bone marrow involvement. A skin biopsy showed diffuse proliferation of atypical cells mainly in the ulcer base and surrounding dermis. A polymorphous population of tumor cells was observed, including large pleomorphic Reed-Sternberg-like cells, small lymphocytes, and immunoblast-like cells. Widespread areas of necrosis were seen throughout the dermis and adipose tissue. Immunohistochemistry showed that the large atypical cells were positive for CD20 and CD30, and negative for CD3, CD5, and CD15. In situ hybridization study revealed that EBV-encoded small RNA (EBER) was positive for atypical lymphocytes. Based on the clinical and histological appearance, a diagnosis of EBVMCU was made. Immunosuppressive drugs were changed to corticosteroids, cyclosporine, and everolimus after the diagnosis of EBVMCU. In a follow-up visit two weeks after the change of immunosuppression therapy, no improvement in the skin ulceration was observed. The patient began radiation therapy with a course of 4 Gy in two fractions over six days using an 8 MeV electron beam with a 5 mm bolus. The irradiation field covered the ulceration and surrounding redness (Figure 1B).

**Figure 2:**
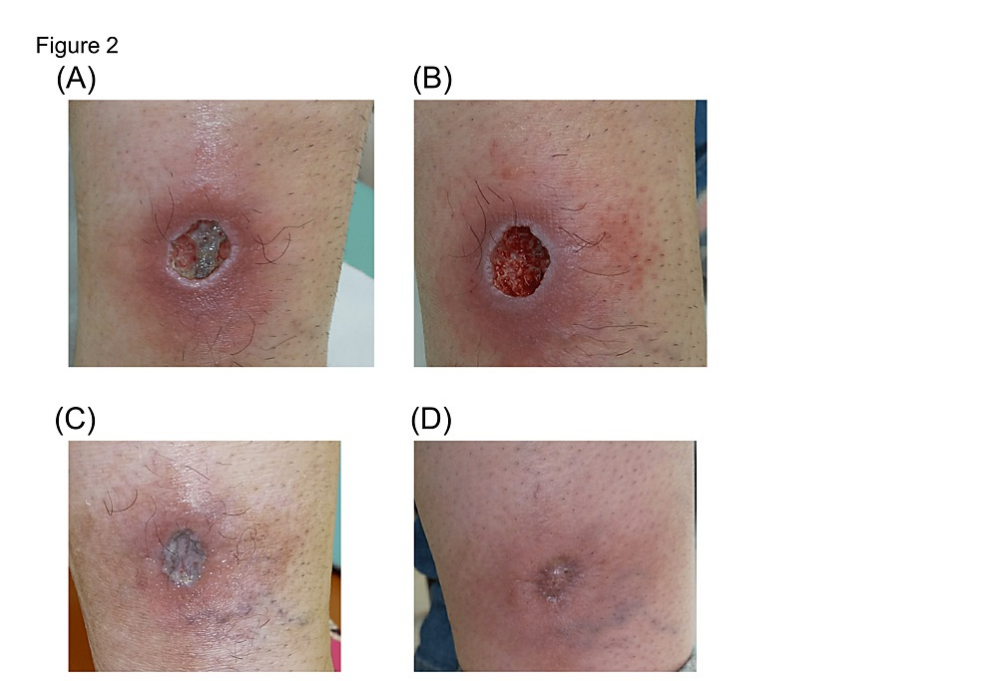
Clinical photograph of the ulcer after treatment: four weeks (A), six weeks (B), eleven weeks (C), and eight months (D).

After two weeks of treatment, no obvious improvement was observed. After four weeks, mild granulation tissue was observed at the ulcer base (Figure 2A). After six weeks, the edge of the lesion became slightly dryer and the depth of the ulcer became shallower (Figure 2B). After 11 weeks, the ulcer was mostly epithelialized (Figure 2C). After one year, all ulcers had undergone epithelialization and a thin crust remained. The patient achieved a complete clinical response. However, redness and induration were present in the surrounding area (Figure 2D). No adverse effects of radiotherapy were noted during follow-up. The patient received no other treatment for EBVMCU after radiotherapy, and continued to take immunosuppressive drugs (corticosteroids, cyclosporine, and everolimus).

## Discussion

A standardized treatment for EBVMCU has not been established to date. EBVMCU is a relatively benign condition that occasionally does not require treatment [8]. Localized EBVMCU lesions are treated by observation or by changing the immunosuppressive therapy regimen [9]. However, in patients who show no improvement with conservative therapies, more aggressive treatment should be considered. Isnard et al. reported that two patients who developed EBVMCU after renal transplantation were treated with rituximab with a curative effect [10]. In the present case, the change in immunosuppressive therapy was not effective, and the patient was subsequently treated with radiotherapy, although the optimal dose and fractionation have not been established. Few reports describe the details of radiotherapy for EBVMCU, including dose fractionation. Roberts et al. reported that a dose of 30 Gy in 15 fractions resulted in a complete clinical response for EBVMCU [7]. This dose is used for other lymphoproliferative conditions such as low-grade lymphoma. However, some patients experience mild toxicities under this radiotherapy schedule. For benign diseases, the risk of late adverse events, including radiation-induced cancer, should be minimized, and the dose and target should be as small as possible.

The strategy in the present case consisted of initial low-dose radiotherapy, followed by an increase in the dose if there was no improvement. We initially planned to use 4 Gy in two fractions, which is a dose used in palliative therapy for low-grade malignant lymphoma. In such patients, complete/partial responses were obtained in 63%-87% of the patient population. A dose of 4 Gy in two fractions rarely causes adverse events [11,12]. In the present strategy, if the lesion did not improve, we planned to add 30 Gy in 15 fractions. At one month after the first radiotherapy course, the lesion did not show marked improvement and the possibility of additional radiotherapy was discussed. Because there are few reports describing the progression of the disease after treatment, it was difficult to determine the optimal timing for deciding whether to provide additional treatment. Since the lesion was relatively benign, we decided to wait, and the patient showed clear improvement after two months. Although the long-term effects remain unknown, at one year after treatment, the 4 Gy dose remains effective.

A randomized trial of follicular and marginal zone lymphoma showed that radiotherapy with 24 Gy achieved better local control than radiotherapy with 4 Gy; the two-year local progression-free rate was 94.1% after 24 Gy and 79.8% after 4 Gy [13]. These findings suggest that the long-term efficacy of the present 4 Gy dose needs to be assessed. Still, because EBVMCU is a relatively benign disease, we believe it is beneficial to consider administering doses that minimize adverse events. In this case, we irradiated not only the ulceration but also the surrounding red areas. However, the surrounding redness remained unchanged after treatment, suggesting that it was an inflammatory response rather than a lymphoproliferative disease. It is unclear whether the irradiated field that included this area would result in local control. The optimal irradiation field needs to be determined.

## Conclusions

EBVMCU is a recently recognized disorder, and a standardized treatment has not been established. Because it is a relatively benign disease, it occasionally does not require treatment. We showed that low-dose radiotherapy was effective in a patient with EBVMCU, and no toxicity was observed. Evaluation to determine further treatment should be performed for at least two months.
